# Anti-Mullerian hormone as a predictor of ovarian reserve in ART protocols: the hidden role of thyroid autoimmunity

**DOI:** 10.1186/s12958-015-0103-3

**Published:** 2015-09-21

**Authors:** Flavia Magri, Lucia Schena, Valentina Capelli, Margherita Gaiti, Francesca Zerbini, Emanuela Brambilla, Mario Rotondi, Mara De Amici, Arsenio Spinillo, Rossella E. Nappi, Luca Chiovato

**Affiliations:** Unit of Internal Medicine and Endocrinology, IRCCS Foundation S. Maugeri, University of Pavia, via S.Maugeri 10, 27100 Pavia, Italy; Department of Pediatrics, University of Pavia, IRCCS Policlinico S. Matteo, Pavia, Italy; Research Center for Reproductive Medicine, Unit of Obstetrics and Gynecology, IRCCS S. Matteo Foundation, University of Pavia, Pavia, Italy

**Keywords:** Anti-Mullerian hormone, Thyroid autoimmunity, Ovarian hyper-stimulation

## Abstract

**Background:**

Protocols of controlled ovarian hyper-stimulation (COH) require, as a crucial step, the identification of reliable predictors of ovarian reserve. Anti-Mullerian hormone (AMH) is one of the most reliable predictors of ovarian reserve but other factors including autoimmune thyroid diseases (ATD) have been associated with reduced fertility and poor COH outcome. Aim of the present study was to evaluate the relationship between ATD and AMH, and their role on the outcome of COH.

**Methods:**

The study group included 288 sub-fertile euthyroid women aged less than 40 years attending a single center for Reproductive Medicine. Among them, 55 were ATD-positive and 233 ATD-negative. The serum levels of AMH, FSH, LH, estradiol (E2), and TSH were measured before COH. The ratio between serum E2 concentration on the day of oocytes pick-up and the total dose of administered recombinant FSH (r-FSH) (E2/r-FSH ratio) was calculated.

**Results:**

The serum levels of AMH were significantly related to E2/r-FSH ratio, total dose of r-FSH and number of M II oocytes, both in ATD-positive and ATD-negative women. Within the low stratum of AMH levels, the presence of ATD did not further affect the outcome of COH. When the serum levels of AMH were in the high stratum, the presence of ATD significantly affected the E2/rFSH ratio, the total dose of r-FSH and the number of M II oocytes.

**Conclusions:**

The probability of a poor response to COH is high, and independent from ATD, in women with low AMH serum levels. In women with a good ovarian reserve, as assessed by high AMH serum levels, the presence of ATD impairs the outcome of COH.

## Background

In developed countries, about 10–15 % of all couples experience difficulties to conceive (primary infertility) or to conceive the number of children they want (secondary infertility) [1]. As a consequence, there is a continuous increase in the number of women undergoing assisted reproductive technologies (ART) in order to achieve a pregnancy. The success rate of ART is influenced not only by the woman’s age and by infertility due to male factors, but also by several biochemical and anatomical variables, which include ovarian response to gonadotropin stimulation, oocyte *in vitro* fertilization, embryonic development and implantation, anatomical alterations of the fallopian tubes and the uterus, endometrial receptivity, and thrombophilic status. All these factors explain the variable response to ART even among women of similar age [2].

Controlled ovarian hyper-stimulation (COH) is a crucial step in all ART protocols. The magnitude of the ovarian response and the number of embryos/blastocysts available for transfer is generally considered to be a function of the gonadotropin dose [3], the type of stimulation protocol [4] and the patient’s profile [2, 5], mainly in terms of age and ovarian reserve. [6, 7]. The identification of reliable predictors of a good ovarian reserve (and as consequence of a favorable outcome) remains a challenge [2]. In the course of ART, a poor ovarian response (POR) to COH is defined by the presence of at least two of the following three features: (i) older maternal age or any other risk factor for POR; (ii) a previous POR; and (iii) an abnormal ovarian reserve test [8]. Tests for ovarian reserve provide an indirect estimate of the cohort of recruitable antral follicles in the FSH window at the beginning of each menstrual cycle [9]. Both the anti-Mullerian hormone (AMH) and the antral follicular count (AFC) are considered reliable and accurate indexes of ovarian reserve [10, 11]. However, as compared to AFC, the measurement of serum AMH has several advantages: a lower intra- and inter-cycle variation [12], the independence from observers’ biases, and the possibility to be used in any clinical setting. For these reasons, AMH is widely recognized as a reliable biomarker of ovarian response [12]. AMH is a member of the transforming growth factor beta family being produced in the granulosa cells of the ovarian follicle [13]. The highest levels of AMH are detected in secondary, pre-antral, and small (up to 6 mm in diameter) antral follicles [14], whereas the production of this hormone ceases in growing to dominance follicles [15, 16]. AMH is barely detectable at birth, peaks after puberty, then decreases progressively with age and becomes undetectable at menopause [17, 18]. In women undergoing COH, the higher the serum levels of AMH, the better is the ovarian response to r-FSH [19].

In addition to the ovarian reserve, which is the main determinant for a good response to COH and for the subsequent ART outcome, autoimmune thyroid diseases (ATD), which are highly prevalent in women during the childbearing age (5–15 %), and hypothyroidism are also associated with reduced fertility and ART failure [20–22]. The relationship between ATD and reduced ovarian reserve has been widely investigated [23–26]. In a previous study, we found that ATD-positive women, as compared to the ATD-negative ones, have a poorer ovarian response to gonadotropins, but data regarding ovarian reserve in these two groups of patients were not investigated [27]. Recently, Kuroda et al. reported that the presence of ATD and the occurrence of a raised TSH (the latter indicating hypothyroidism) were associated with low serum levels of AMH [28]. In another study, a significant association was found between ATD and low serum levels of AMH, which was independent from age [29]. At variance with these reports, other authors found that serum AMH levels were significantly higher in women with ATD compared with a control group [30]. Moreover, in a large cross-sectional analysis, Polyzos et al. reported that ATD and hypothyroidism were not associated with a reduced ovarian reserve, as assessed by low age-specific AMH values [31].

Aim of the present study was to investigate the relationship between thyroid autoimmunity and ovarian reserve, as assessed by serum AMH concentrations, and to evaluate their respective role on the outcome of COH, being defined as ovarian response to recombinant gonadotropins.

## Methods

### Study group

In a retrospective, single-center study, we evaluated a cohort of patients undergoing their first ART procedure from January 2011 and April 2014. The study group included 288 euthyroid (serum TSH level < 2.5mIU/L according to the current guidelines [32–35]) women aged less than 40 years with no history of previous POR and no evidence of other risk factors, both genetic and acquired. Among them, clinical and/or biochemical evidence of ATD, as assessed by positive tests for anti-thyroglobulin and/or anti-thyroid-peroxidase antibodies (serum levels at least two times higher than the reference range), was present in 55 patients (the ATD-positive group) and absent in the remaining 233 (the ATD-negative group). In the former group, 28 (50.9 %) women were euthyroid on L-Thyroxine (LT4) substitution treatment. All subjects were Caucasian women resident in a Northern Italy area, which is characterized by mild iodine deficiency. The following inclusion criteria were used: i) sub-fertility of various cause; ii) indication for an ART cycle through a long GnRH agonist protocol with r-FSH; iii) a euthyroid state at the time of COH, either spontaneous or resulting from LT4 substitution treatment; iv) information about tests for thyroid autoimmunity. Before starting COH, serum TSH, AMH, FSH, LH, estradiol (E2) and PRL at cycle day 3, and serum progesterone (P) at cycle day 21 were measured.

The study was approved by the local Institutional Board and all patients gave their written informed consent concerning the future use of clinical and hormone data for research purposes according to the local Ethics Committee and the guidelines of the Declaration of Helsinki.

### COH protocol

Ovarian suppression was achieved by administering subcutaneously a daily dose (1 mg) of leuprorelin acetate (Enantone, Takeda Italia Farmaceutici S.p.A, Italy) started on day 21 of the previous cycle. Both ultrasound scan (absence of ovarian activity, ovarian cyst formation and endometrial proliferation) and serum E2 levels (≤40 pg/mL) confirmed ovarian suppression before starting exogenous gonadotropin administration for COH. A fixed daily dose (150 IU) of r-FSH (Gonal-F; Merck-Serono, Rome, Italy) was used for 5 days, until the ovarian response was evaluated, and then adjusted accordingly. Starting from day 9, women were observed daily until criteria were fulfilled for discontinuing the GnRH agonist and for administering hCG at a dose of 10,000 IU i.m.. Ovulation was triggered by hCG treatment when at least two leading follicles, with a mean diameter >18 mm, were detected. An adequate rise of serum E2 concentration was observed during COH. No longer than 24 h elapsed from the last injection of r-FSH and the administration of hCG. Thirty-five to 36 h after hCG administration, ovum pick-up was performed by trans-vaginal ultrasound-guided ovarian puncture. After stripping, oocytes were assessed for their maturation; only oocytes having resumed their first meiotic division and reaching metaphase II were used for IVF. On the day of transfer, embryos were graded according to their morphologic appearance under a light microscope and then transferred.

The ratio between serum E2 concentration on the day of pick-up and the total number of r-FSH units administered during the CHO procedure (E2/r-FSH ratio) was calculated. This allowed measuring the performance of r-FSH in producing an adequate ovarian stimulation in ATD-positive as opposed to ATD-negative women

### Serum assays

The serum concentrations of TSH (third generation TSH assay; normal range 0.4–4 mIU/L) were measured using an immune-chemiluminescent assay run by an automated analyzer ((Immulite 2000, DPC Cirrus, Los Angeles, CA, USA) employing commercial kits (Diagnostic Products Corporation, Los Angeles, CA, USA). The sensitivity of the assay was 0.004 mIU/L and the intra- and inter-assay coefficients of variation were 3.5 and 5.4 %, respectively. The serum concentrations of anti-thyroglobulin antibody (Tg-Ab; normal range <60 U/mL) and anti-thyroid peroxidase antibody (TPO-Ab; normal range < 60 U/mL) were measured using immune-chemiluminescent assays employing commercial kits (Brahams, Hennigsdorf, Germany). The sensitivity of the assay was 33 U/mL for TG-Ab and 50 U/mL for TPO-Ab. The intra- and inter-assay coefficients of variation were 2.6 and 13 % respectively, for TG-Ab and 3.9 and 8 %, respectively, for TPO-Ab.

The serum levels of estradiol (E2) and progesterone (P) were measured using a commercial RIA kit (Diagnostic Systems Laboratories, Inc.). The sensitivity of the assay was 2.2 pg/mL and 0.12 ng/mL, respectively; the intra- and inter-assay coefficients of variation were 7.5 and 9.3 %, for serum E2 and 6.8 and 8.8 %, for P respectively. FSH and LH, as well as PRL, were measured by immune-enzymatic assays (FSH and PRL, Abbott Laboratories, Rome, Italy; LH, Dade Behring, Milan, Italy). The sensitivity of the assay was 0.2 U/L for FSH and LH; the intra- and inter-assay coefficients of variation were: FSH, 4.7 and 8.9 %, respectively; LH, 3.1 and 4.0 %, respectively. The sensitivity of the assay was 0.6 ng/mL for PRL; the intra- and inter-assay coefficients of variation were 5.3 and 7.1 %, respectively.

During the study period, the serum concentrations of AMH were measured by an enzymatically amplified two-site immunoassay (AMH Gen II ELISA, Beckman Coulter, Brea, CA, USA) [36, 37]. Normal values for AMH were < = 12.6 ng/mL. The analytical sensitivity of the assay was 0.08 ng/mL and the intra- and inter-assay coefficients of variation were 5.4 and 5.6 %, respectively, according to the products’ inserts. The lowest amount of AMH in a sample that could be detected with a 95 % probability was 0.08 ng/mL. The estimated minimum dose achieved at 20 % total imprecision was 0.16 ng/mL.

### Statistical analysis

Quantitative values were expressed as mean and 95 % confidence interval or as median and interquartile range, as appropriate. Between-group comparisons were performed by Student’s *t*-test for unpaired data and by the Mann–Whitney U test according to a normal or a non-parametric distribution of data. When more than two groups were compared, the analysis of variance (Kruskal-Wallis ANOVA) was performed. The coefficient of correlation was calculated with the Spearman Rank Order Correlations. A p value <0.05 was considered statistically significant. Statistical analysis was performed using the SPSS software (SPSS, Inc., Evanston, IL, USA).

## Results

Table 1 shows the clinical and biochemical data of sub-fertile women included in the study, divided according to their ATD status. The ATD-positive and -negative groups were similar for age, body mass index (BMI), cause of infertility, serum TSH levels and serum concentrations of E2, PRL and LH in the early follicular phase. Also similar were the serum concentrations of progesterone during the luteal phase preceding the ART procedure. The serum levels of AMH and FSH on day 3 were significantly higher and significantly lower, respectively, in ATD-negative women compared with the ATD-positive ones.

**Table 1:** Clinical and hormonal features of sub-fertile women divided according to their autoimmune thyroid (ATD) status. Results are expressed as mean (95 % confidence interval) or percent of the corresponding group

	ATD negative	ATD-positive	*P*
	*N* = 233	*N* = 55	
Age (years)	34.64 (33.1–34.1)	35.8 ± 3.62 (34.9–36.8)	0.106.
BMI (kg/m^2^)	22.62 (22.1–23.1)	21.91 (21.1–22.6)	0.174
PCOS (%)^a^	9.3	7.3	0.613
Endometriosis (%)	15.8	7.27	0.114
Male factor (%)	30.9	36.4	0.434
AMH (ng/mL)	2.27 (1.7–2.3)	1.40 (1.0–1.7)	0.034
TSH (mIU/L)	1.86 (1.7–1.9)	1.96 (1.7–2.2)	0.536
FSH (IU/L)^b^	7.96 (7.4–8.4)	9.57 (8.1–11.0)	0.009
LH (IU/L)^b^	6.78 (4.1–9.4)	6.80 (4.9–8.6)	0.994
Estradiol (pg/mL)^b^	61.40 (55.5–67.3)	70.65 (51.8–89.4)	0.227
Prolactin (ng/mL)^b^	17.26 (15.3–19.8)	15.60 (13.3–17.9)	0.442
Progesterone (ng/dL)^c^	11.8 (10.4–13.1)	9.48 (7.3–11.5)	0.107

^a^PCOS: Polycystic Ovary Syndrome

^b^on cycle day 3

^c^on cycle day 21

The mean total dose of administered r-FSH did not significantly differ between ATD-negative and ATD-positive women (2273.8 IU, 95 % CI 2082–2465 and 2298.4 IU, 95 % CI 2034–2562, respectively). The E2/r-FSH ratio at oocyte pick-up was significantly lower in ATD-positive women compared with the ATD-negative ones (0.57, 95 % CI 0.44-0.70 and 1.05, 95 % CI 0.82-1.22, respectively, *p* = 0.026). This finding indicates a poorer performance of r-FSH in producing ovarian stimulation in ATD-positive women.

## AMH and COH outcome

**Fig. 1:**
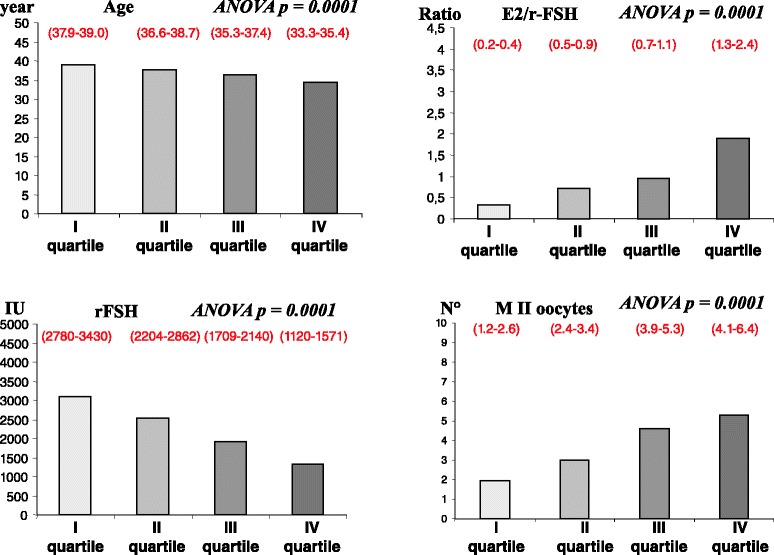
quartiles of distribution of serum AMH. Age, E2/r-FSH ratio, total dose of administered r-FSH and number of MII oocytes in subfertile women subdivided according to quartiles of distribution of their serum AMH (first quartile = lowest values, fourth quartile = highest value). Results are expressed as mean and (95 % confidence interval)

The age of sub-fertile women and several parameters indicating the outcome of COH were evaluated according to the stratification in quartiles of the serum levels of AMH. Quartiles were obtained dividing the study group into four equal parts (each including 72 women) (Fig. 1). Quartiles were defined by the following ranges of serum AMH concentration: I = 0–0.5 ng/mL; II = 0.54–1.32 ng/mL; III = 1.33–2.70 ng/mL; IV 2.8–18.2 ng/mL. The first quartile corresponded to percentile 25, the second quartile to percentile 50 and the third quartile to percentile 75. The prevalence of ATD-positive women in each quartile was 20.8, 22.2, 21.9 and 11.4 %, respectively. In the upper quartile of AMH distribution women were significantly younger and required a lower total dose of r-FSH. The performance of r-FSH in stimulating the ovarian secretion of E2 (as assessed by the E2/r-FSH ratio) was significantly better when going from the first to the fourth AMH quartile. A similar trend was found when the number of M II oocytes was considered.

When sub-fertile women were stratified according to the presence or absence of ATD, linear regression analysis confirmed the relationship between the serum levels of AMH and the outcome of COH (as assessed by E2/r-FSH ratio, total dose of r-FSH and number of M II oocytes) both in ATD-positive and ATD-negative women (Table 2).

**Table 2:** Linear regression analysis relating the serum levels of AMH and age or COH outcomes (E2/r-FSH ratio, total dose of r-FSH and number of M II oocytes) in ATD-positive and ATD-negative women

AMH	Age	E2/rFSH	rFSH	M II oocytes
ATD-negative	*R* = −0.236	*R* = 0.628	*R* = −0.582	*R* = 0.388
	*P* = 0.001	*P* = 0.0000	*P* = 0.0000	*P* = 0.0000
ATD positive	*R* = −0.403	*R* = 0.381	*R* = −0.371	*R* = 0.554
	*P* = 0.005	*P* = 0.009	*P* = 0.008	*P* = 0.0000

## ATD, AMH, and COH

The second quartile of AMH distribution, which corresponded to a median serum AMH level of 1.32 ng/mL, was considered the cut-off level to stratify sub-fertile women in a low (serum AMH < 1.32 ng/mL) and a high (serum AMH > 1.32 ng/mL) AMH stratum. According to this cut-off value, the low AMH stratum included older women with higher basal serum levels of FSH. These women, despite receiving a greater dose of r-FSH, had a lower E2/r-FSH ratio and a lower number of M II oocytes (Table 3).

**Table 3:** Clinical and hormonal features, and outcome of COH in sub-fertile women divided according to the median level of serum AMH (low AMH stratum < 1.32 ng/mL and high AMH stratum > 1.32 ng/mL). Results are expressed as mean (95 % confidence interval)

	Low AMH stratum	High AMH stratum	*p*
	<1.32 ng/mL	>1.32 ng/mL	
Age (years)	36.39 (35.6–37.1)	33.39 (32.6–35.1)	0.001
AMH (ng/mL)	0.53 (0.4–0.6)	3.53 (2.9–3.6)	0.001
BMI (kg/m^2^)	22.25 (21.6–22.8)	22.69 (22.1–23.2)	0.284
FSH (UI/L)	9.43 (8.5–10.2)	7.10 (6.7–7.5)	0.001
Estradiol (pg/mL)	66.96 (58.3–75.5)	59.64 (51.3–67.9)	0.226
TSH (mUI/L)	1.84 (1.7–1.9)	1.92 (1.7–2.0)	0.514
r-FSH (UI)	2836.19 (2598.4–3073.9)	1698.27 (1532.5–1864.1)	0.001
Estradiol at oocyte pick-up (pg/mL)	981.05 (853.5–1108.5)	1729.32 (1489.9–1968.5)	0.001
E2/r-FSH ratio	0.53 (0.3–0.6)	1.3 (1.0–1.6)	0.001
Number of MII oocytes	2.51 (2.0–2.9)	4.87 (4.2–5.4)	0.001

**Fig. 2:**
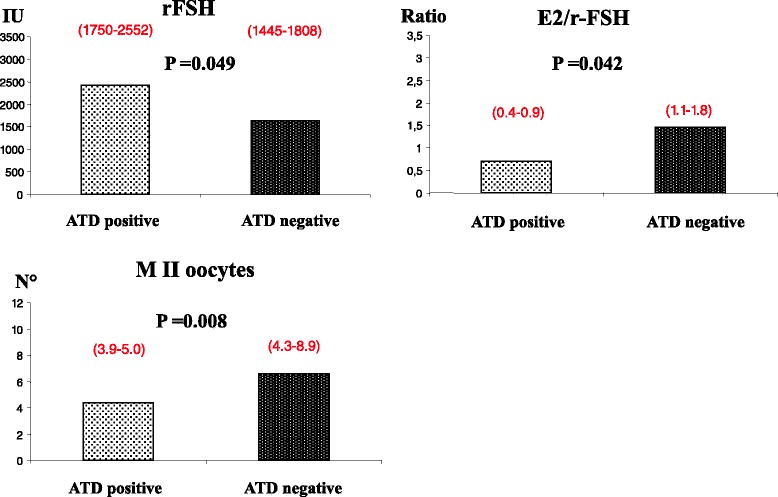
COH outcome according to autoimmune thyroid status and AMH stratum. COH outcome in sub-fertile women included in the high AMH stratum (AMH > 1.3 ng/mL) divided according their autoimmune thyroid (ATD) status. Results are expressed as mean and (95 % confidence interval)

Within the low AMH stratum, the presence or absence of ATD did not further affect the outcome of COH (Table 4). Only the basal serum levels of FSH were significantly higher in ATD-positive women when compared with the ATD-negative ones. In the high AMH stratum (>1.32 ng/mL), the presence of ATD became relevant. Indeed, ATD-positive women showed a lower, although not significant, concentration of serum E2 at oocyte pickup, and a significantly lower E2/rFSH ratio (Fig. 2). The total dose of administered r-FSH was also significantly higher and the number of M II oocytes was significantly lower in ATD-positive compared with ATD-negative women.

**Table 4:** Clinical and hormonal data, and outcome of CHO, in sub-fertile women within the low AMH stratum (AMH < 1.32 ng/mL) divided according their autoimmune thyroid status (ATD) Results are expressed as mean (95 % confidence interval)

	Low AMH stratum	Low AMH stratum	*p*
	ATD-negative	ATD-positive	
Age (years)	36.32 (35.4–37.2)	34.6 (33.2–36.9)	0.753
AMH (ng/mL)	0.52 (0.4–0.6)	0.56 (0.4–0.7)	0.599
BMI (kg/m^2^)	22.41 (21.7–23.1)	22.61 (20.8–22.4)	0.344
FSH (UI/L)	8.84 (7.9–9.7)	11.48 (9.3–13.6)	0.010
Estradiol (pg/mL)	69.67 (59.3–80.0)	58.03 (43.4–72.6)	0.258
TSH (mUI/L)	1.82 (1.6–1.9)	1.92 (1.5–2.2)	0.632
r-FSH (UI)	2948.29 (2658.4–3236.1)	2368.96 (2111.3–2825.3)	0.091
E2 pick (pg/mL)	968.22 (822.0–1114.4)	1019.07 (741.6–1296.4)	0.733
E2/r-FSH ratio	0.54 (0.3–0.7)	0.49 (0.3–0.6)	0.735
N^ MII oocytes	2.61 (2.1–3.1)	2.21 (1.2–2.2)	0.433

## Discussion

Both a reduced ovarian reserve (expressed by low serum levels of AMH) and the occurrence of ATD exert a negative influence on the outcome of COH, which represents a crucial step of ART. Our results confirm that ovarian reserve is the major determinant of ovarian response to COH. Indeed, when serum AMH levels were low, the outcome of COH was poor. The relationship between ATD and ovarian reserve, and the role of ATD by itself on the success of COH, are more intriguing. In the present study, we found significantly lower serum levels of AMH in ATD-positive women compared with the ATD-negative ones. This is in line with some previous findings [29], but in contrast with data from a recent large cross-sectional analysis, in which neither ATD nor hypothyroidism were found to be associated with a poor ovarian reserve, as assessed by low age-specific AMH values [31]. A possible explanation for this discrepancy might be the lack of an age-specific stratification of AMH levels.

ATD represents the main cause of thyroid failure in women of childbearing age [34] and, even when subclinical, it can impair fertility and increase pregnancy-related morbidities [32, 38]. For this reason, current guidelines for thyroid dysfunction in pregnancy [32–35] emphasize the crucial role of preconception serum TSH measurement, and recommend LT4 therapy in women with ATD and associated subclinical hypothyroidism in order to maintain a serum TSH concentration < 2.5 mIU/L [33, 34]. These Guidelines are nowadays widely followed in the clinical management of spontaneous pregnancies, but their application in ART pregnancies is still debated. In this context, we recently demonstrated that the outcome of COH was poorer in ATD-positive compared with ATD-negative women, but it significantly improved when the pre-stimulation TSH was lower than 2.5 mIU/L [27]. Moreover, among ATD positive women, a trend toward a better ovarian response was found in those belonging to the lower quartiles of TSH [27].

In order to investigate the respective role of ovarian reserve and ATD on the outcome of COH, we enrolled euthyroid women (TSH <2.5mIU/L) with no previous POR or any other risk factor, both genetic and acquired. All other conditions possibly affecting COH, such as BMI, PCOS and endometriosis [39, 40], were equally distributed in the different groups of sub-fertile women. To assess the outcome of COH, we evaluated the total dose of administered r-FSH, the r-FSH performance (i.e. the E2/r-FSH ratio) [27], and the number of retrieved MII oocytes [41]. ATD-positive women showed a lower E2/r-FSH ratio, confirming a negative role of ATD on the outcome of COH [27]. However, this negative influence of ATD was observed only in women with a preserved ovarian follicle reserve, as assessed by their high serum levels of AMH. In women with low serum levels of AMH, the outcome of COH was invariably poor and independent from their ATD status. As expected, higher basal serum levels of FSH were found in women belonging to the low AMH stratum. Similar findings were observed in ATD-positive women. These data confirm the negative predictive value of high serum FSH levels on the outcome of ART [42].

FSH receptor polymorphisms were not evaluated in the present study. This represents a possible limitation because previous data suggest that FSH receptor variants might influence the ovarian response to r-FSH during COH [43].

## Conclusions

The present data further support the recommendation of assessing both ovarian reserve and thyroid autoimmunity when planning an ART procedure. In women with low serum AMH levels the probability of a successful outcome is low, independently from thyroid autoimmunity. In women with a preserved ovarian reserve, the presence of ATD impairs the outcome of CHO, at least when standard doses of recombinant gonadotropins are used. In this view, a pre-stimulation screening for thyroid autoimmunity appears mandatory even in euthyroid women.
